# Supplementary material for: Identification of photoperiod-induced specific miRNAs in the adrenal glands of Sunite sheep (Ovis aries)
Source: Front Vet Sci. 2022 Jul 22;9:888207. doi: 10.3389/fvets.2022.888207 (PMC9354845; doi:10.3389/fvets.2022.888207)
Supplement: Supplementary file 1 [file Data_Sheet_1.ZIP › Supplementary file/miRDeep2 file.zip!/miRDeep2 file/Know_Structure/SP42A2/oar-mir-3957.pdf]

miRBase precursor : oar-mir-3957
Total read count : 125
oar-miR-3957-5p read count : 90
oar-miR-3957-3p read count : 34
remaining reads : 1

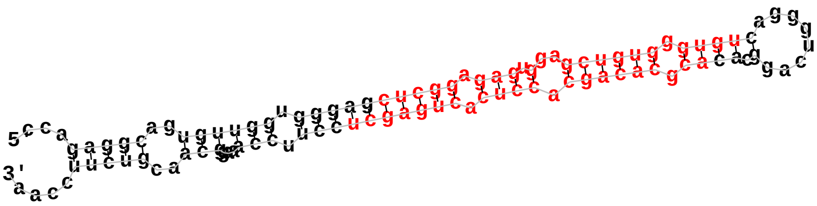

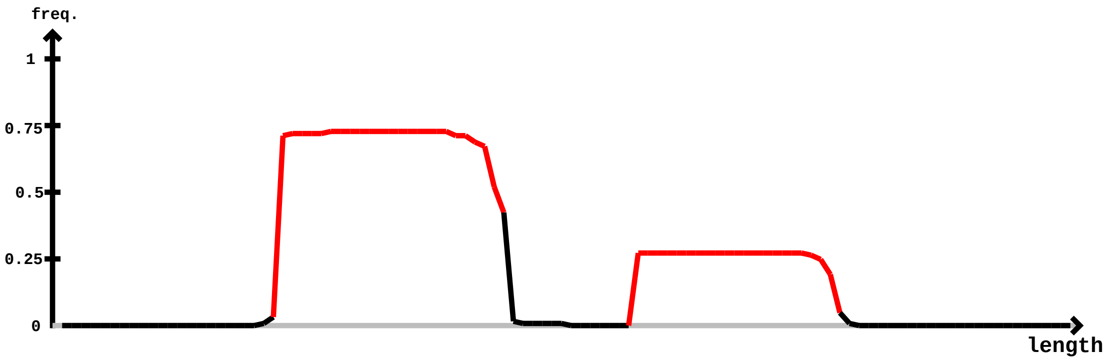

oar-miR-3957-5p

oar-miR-3957-3p

```
5'- ccagaggcaguguuggugggagcucggagaguggagcugugggugucagggucaggcacacgcacagcaccucacugagcuccuuccaaaggcaacgucuuccaa   -3'    exp
    ...(((((..((((((.((((((((((.(((.(..((((((.(((((........).)))).)))))).)))).)))))))))).)))...)))..)))))....   reads   mm   sample
    ....................agcucggagaguggagcugugggugu...........................................................   1       0    seq
    .....................gcucggagaguggagcugugggu.............................................................   1       0    seq
    .....................gcucggagaguggagcugugggug............................................................   2       0    seq
    ......................cucggagaguggagcugu.................................................................   2       0    seq
    ......................cucggagaguggagcugugg...............................................................   3       0    seq
    ......................cucggagaguggagcuguggg..............................................................   2       0    seq
    ......................cucggagaguggagcugugggu.............................................................   18      0    seq
    ......................cucggagaguggagcugugggug............................................................   9       0    seq
    ......................cucggagaguggagcugugggugu...........................................................   50      0    seq
    ......................cucggagaguggagcuguggguguc..........................................................   1       0    seq
    .......................ucggagaguggagcugugggug............................................................   1       0    seq
    ...........................agaguggagcugugggugucagggu.....................................................   1       0    seq
    ...........................................................acgcacagcaccucacug............................   1       0    seq
    ...........................................................acgcacagcaccucacuga...........................   2       0    seq
    ...........................................................acgcacagcaccucacugag..........................   7       0    seq
    ...........................................................acgcacagcaccucacugagc.........................   18      0    seq
    ...........................................................acgcacagcaccucacugagcu........................   5       0    seq
    ...........................................................acgcacagcaccucacugagcuc.......................   1       0    seq
```
